# Algorithms of follow-up in patients with head and neck cancer in relation to primary location and advancement. Consensus of Polish ENT Society Board and Head Neck Experts

**DOI:** 10.3389/fonc.2023.1298541

**Published:** 2023-12-12

**Authors:** Małgorzata Wierzbicka, Jarosław Markowski, Wioletta Pietruszewska, Paweł Burduk, Bogusław Mikaszewski, Marek Rogowski, Krzysztof Składowski, Piotr Milecki, Jacek Fijuth, Dariusz Jurkiewicz, Kazimierz Niemczyk, Adam Maciejczyk

**Affiliations:** ^1^ Department of Otolaryngology, Regional Specialist Hospital Wroclaw, Research & Development Centre, Wroclaw, Poland; ^2^ Faculty of Medicine, Wroclaw University of Science and Technology, Wroclaw, Poland; ^3^ Institute of Human Genetics, Polish Academy of Sciences, Poznan, Poland; ^4^ Department of Laryngology, Faculty of Medical Sciences in Katowice, Medical University of Silesia in Katowice, Katowice, Poland; ^5^ Department of Otolaryngology Head Neck Oncology, Medical University of Lodz, Lodz, Poland; ^6^ Department of Otolaryngology Phoniatrics and Audiology, Nicolaus Copernicus University in Toruń, Bydgoszcz, Poland; ^7^ Department of Otolaryngology, Faculty of Medicine, Medical University of Gdansk, Gdansk, Poland; ^8^ Department of Otolaryngology, Medical University of Bialystok, Bialystok, Poland; ^9^ Radiation and Clinical Oncology Department, Maria Skłodowska-Curie National Research Institute of Oncology, Gliwice Branch, Gliwice, Poland; ^10^ Department of Radiotherapy I, The Greater Poland Cancer Centre, Poznan, Poland; ^11^ Department of Radiation Therapy, Oncology Chair, Medical University of Lodz, Lodz, Poland; ^12^ Department of Otolaryngology and Laryngological Oncology with Clinical Department of Cranio-Maxillofacial Surgery, Military Institute of Medicine - National Research Institute, Warsaw, Poland; ^13^ Department of Otorhinolaryngology Head and Neck Surgery, Medical University of Warsaw, Warsaw, Poland; ^14^ Department of Oncology, Wroclaw Medical University, Wroclaw, Poland

**Keywords:** algorithm, head and neck cancer, follow-up, salvage, risk of failure, primary location, advancement

## Abstract

**Summary:**

The algorithm of follow-up in patients with head and neck cancer (HNC) has been prepared by a board of Polish Head Neck and Oncology Experts. The aim of this research is to focus on the specificity of HNC monitoring, to review the current trends in follow-up, and to adapt the evidence-based medicine international standards to the capabilities of the local healthcare service.

**Materials and methods:**

The first methodological step was to categorize HNCs according to the estimated risk of failure after the adequate first-line treatment and according to the possibility of effective salvage treatment, resulting in improved overall survival. The final method used in this work was to prepare an authors’ original monitoring algorithm for HNC groups with a high, moderate, and low risk of recurrence in combination with a high or low probability of using an effective salvage.

**Results:**

Four categories were established: Ia. low risk of recurrence + effective organ preservation feasible; Ib. low risk of recurrence + effective salvage feasible; II. moderate risk of recurrence + effective salvage feasible; III. high risk of recurrence + effective salvage feasible; and IV. high risk of recurrence + no effective salvage feasible. Follow-up visit consisting of 1. ENT examination + neck ultrasound, 2. imaging HN tests, 3. chest imaging, 4. blood tests, and 5. rehabilitation (speech and swallowing) was scheduled with a very different frequency, at the proposed monthly intervals, tailored to the needs of the group. The number of visits for individual groups varies from 1 to 8 in the first 2 years and from 1 to 17 in the entire 5-year monitoring period. Group IV has not been included in regular follow-up, visits on own initiative of the patient if symptomatic, or supportive care needs, having in mind that third-line therapy and immune checkpoint inhibitors are available.

**Conclusion:**

Universal monitoring algorithm for HNC four groups with a high, moderate, and low risk of recurrence after the adequate treatment in combination with a high or low probability of using an effective salvage is an innovative approach to redeploying system resources and ensuring maximum benefit for patients with HNC.

## Introduction

- The presented algorithms of follow-up in patients with head and neck cancer (HNC) are complementary to the basic document, which is the National Comprehensive Cancer Network (NCCN) Clinical Practice Guidelines, in which, however, post-treatment monitoring is not an extensive topic.
- These algorithms have a practical focus and are a handy document for a wide range of otolaryngologists who are responsible for monitoring patients after treatment, often outside leading oncological centers.
- The novel approach is the categorization of all patients with HNC, regardless of primary location. All HNCs are classified into one of the four groups, based on the estimated risk of failure after first-line treatment and the possibility of applying an effective salvage associated with improved survival.
- On the basis of this assumption, four different monitoring algorithms were developed for HNCs, classified as low, medium, and high risk of recurrence and palliative group. Accordingly, there are four further follow-up paths to practical application.

Follow-up is a well-established method of care for oncological patients. It is a common practice for most types of cancer to monitor survivors after treatment (1–5). Up to date, there is no consensus on the optimal duration of post-treatment follow-up after HNC (6), although monitoring standards, outside the NCCN, have been provided by the following societies around the world: ASHNS (American Society for Head and Neck Society, 2016), SHNS (Society of Head and Neck Surgery, 1999), BAHNO (British Association of Head and Neck Oncologists, 2001), DAHANCA (Danish Head and Neck Cancer Group, 2013), and ELS (European Laryngological Society, 2014) - Head and Neck Cancer Committee Working Group (7).

This algorithm is prepared by a board of Polish Head Neck and Oncology Experts. The aim is to 1. focus on the specifics of HNC follow-up and review the current world trends; 2. generate input data for an innovative approach to create new, simplified guidelines by categorizing the risk of recurrence and the chance for salvage for all HNCs; and 3. adapt the created algorithm to international standards and the capabilities of the local health service.

### Definition for post-treatment monitoring

Follow-up is a long-lasting and regular maintenance of contact with and re-examination of a patient, especially following oncological treatment, at specified intervals, in a medically documented manner and using specified technical means.

### Rationale for post-treatment monitoring

Patient monitoring programs are based on the assumption that a possible asymptomatic recurrence will be characterized by a lower stage of advancement recurrent tumour, node (rTN) than in the case of a patient reporting due to the appearance of ailments. If a diagnosis is made during a routine, scheduled visit, then there is a greater chance of implementing effective salvage therapy.

### Outcome measures for post-treatment monitoring

Success in monitoring patients with cancer is firstly measured by the percentage of detected recurrences that can be qualified for subsequent radical treatment. Other measures include the percentage of effective salvage treatment and higher disease-related survival (5, 6, 8). Extending the patient’s survival time on the scale of the entire observed population reduces the number of disease-related deaths.

### The specificity of HNC follow-up

However, for HNC, long-term routine follow-up remains a matter of debate (9–13). The benefits of routine follow-up for patients with HNC measured by extending the patient’s survival time have not been proven (14–18). Nevertheless, there are some additional issues bound with follow-up ideas (14, 19). Monitoring also aims to 1. assess the effectiveness of treatment; 2. early diagnose and treat complications, therapy failures, and sequelae (20); 3. detect a second primary tumor (SPT) (19, 21–23); and 4. implement psychological care and provide the patient with constant contact with the center, where he was treated (24–28).

### Premises for logistics, cost-effectiveness, and maximization of health benefit

Organization of efficient outpatient care for patients with HNC constitutes a huge organizational challenge. Post-treatment monitoring puts increasing pressure on healthcare resources due to rising morbidity and, at the same time, rising survival rates. Therefore, many centers are analyzing a new approach to the observation of patients with HNC, giving them the opportunity to choose their own monitoring program (5, 6, 29). Determining the optimal schedule of follow-up visits for assessment for patients with HNC depends on multiple factors.

The first group of variables depends on the characteristics of the tumor. It is stratified by primary location, baseline tumor, node, metastases (TNM) stage, associated risk of recurrence, histological features of the tumor (e.g., Ki67), Human Papilloma Virus (HPV) status, final histological result with assessment of risk factors (R0, R1, R2, and tumor cell emboli in the microscopic vessels, perineurium infiltration, and extracapsular spread), and concepts and methods of treatment (surgical/radiotherapy/radiochemotherapy (RT/RTCT), definitive, palliative, and supportive).

The second group of variables depends on the patient status. The patient’s willingness to cooperate with medical staff, compliance, and checkup visits’ attendance should be of note. It is often a derivative of the distance from the treatment center, communication possibilities, age, education, health awareness, general condition, and cooperation with the family. Well-established risk factors for recurrence include older age, site of primary, male sex, smoking habit, and negative HPV status (30). Some prognostic factors are also predictors. HPV positivity indicates a better response to chemioradiotherapy (CRT). An advanced age is bound with a worse response, and elderly are usually not fit for re-irradiation or salvage surgery (16, 18).

The third group of variables depends on the capabilities of the treatment and monitoring center. This includes human resources, premises, access to imaging tests, and organizational efficiency in coordinating patient monitoring. The IT (Information Technology) infrastructure, efficient communication, and media (phones, text messages, and e-mails) availability are crucial.

Because there is no clear answer on how efficient and cost-effective monitoring is and what the real chances for recurrence, metastasis, or second primary cancer cure are, innovative rules of follow-up have been introduced. Salvage with the intention of radical curation is possible in less than 50% of monitored patients and applies only to those initially treated in the early stages of cancer. That is, the greatest therapeutic benefit is achieved by selected patients, in whom reducing the intensity of monitoring, i.e., limiting the number of visits, could jeopardize the patient’s chances of re-treatment with the organ preservation intent. Patients with extensive cancer have the lowest profit; even after early detection of recurrence, it is difficult to suggest any further treatment option in this group (6). The monitoring algorithms for individual primary locations and cancer stages according to the Cohort Study with Parametric Modelling of Event-Free Survival by Lee et al. (5) was the quintessence of a new therapeutic approach and a starting point for the development of Polish HNC follow-up recommendations (31).

## Method

The first methodological step was to categorize HNCs according to the estimated risk of failure after the adequate first-line treatment and according to the possibility of effective salvage treatment associated with improved survival. The use of a prognostic model for patients with HNC receiving care at medical centers in developed countries was recommended and followed in the methods section (online at http://www.oncologiq.nl).

The final method used in this work was to prepare an authors’ original monitoring algorithm for HNC groups with a high, moderate, and low risk of recurrence in combination with a high or low probability of using an effective salvage.

**Figure 1:**
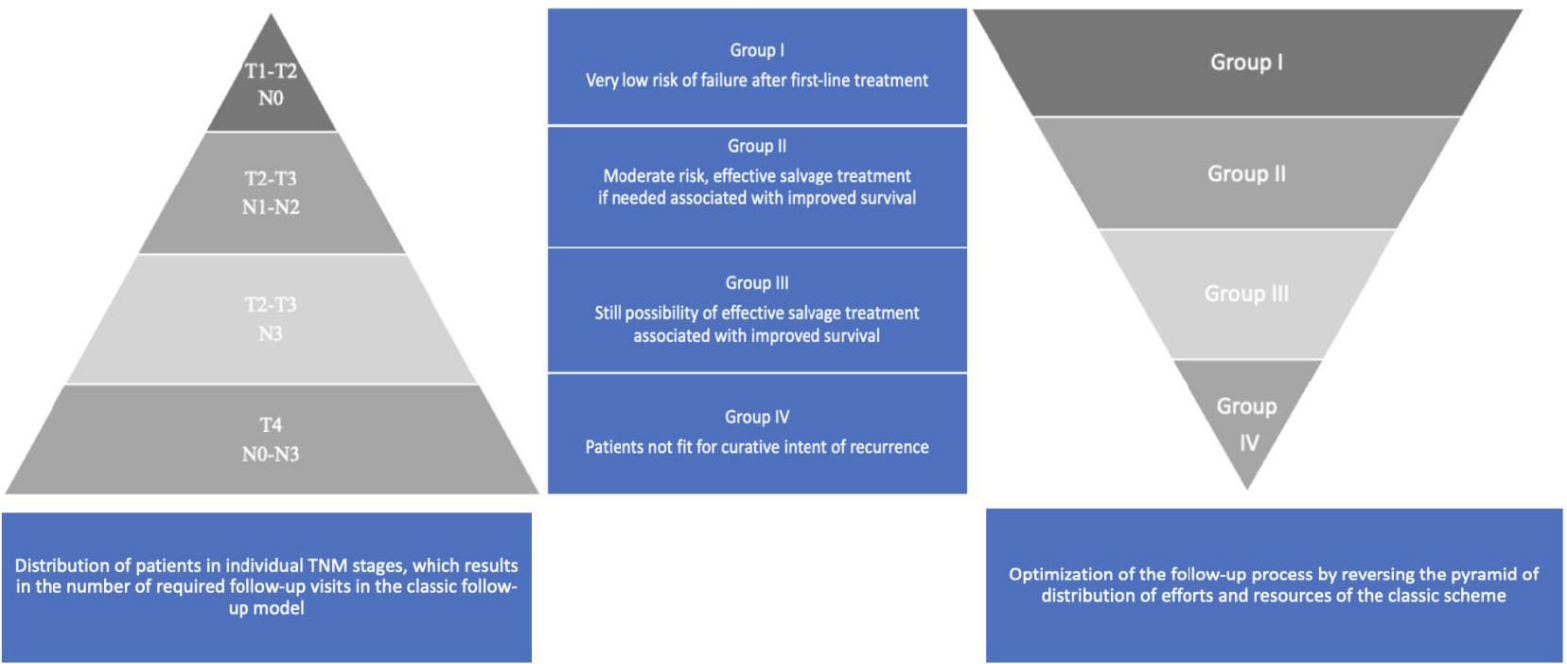
HNCs grouped according to the estimated risk of failure after first-line treatment and according to the possibility of effective salvage treatment associated with improved survival (online at http://www.oncologiq.nl). RT - Radiotherapy alone, RT/CT - Radio chemotherapy. ↑- upgrade to the higher group if there are adverse prognostic factors, increasing the risk. ^1^Larynx - Anterior Commissure, Posterior Paraglottic space involvement,. ^2^Larynx - prior tracheotomy, ^3^ Larynx - N advancement. ^4^ Larynx - N advancement, prior tracheotomy. ^5.6^ Patient treated as negative if the EBV, HPV status unknown.

The main research assumption and the premise for creating recommendations is to answer a question on how to optimize the HNC follow-up and is shown in **Figure 1**.

## Results

The monitoring algorithms for distinct primary locations—larynx, nasopharynx, oropharynx, hypopharynx, oral cavity, and particular cancer stages (TNM)—were created. The primary treatment modality was taken into consideration as well as second- and third-line treatment capabilities. Except the malignant sinonasal and salivary gland tumors, the common benign entities, such as inverted papilloma (IP) and pleomorphic adenoma (PA) follow-up schedules, were also enlisted.

Four categories of HNC were established: Ia. low risk of recurrence + effective organ preservation feasible; Ib. low risk of recurrence + effective salvage feasible; II. moderate risk of recurrence + effective salvage feasible; III. high risk of recurrence + effective salvage feasible; and IV. high risk of recurrence + no effective salvage feasible (**Tables 1A–F**).

**Table 1a:** HNCs grouped according to the estimated risk of failure after first-line treatment and according to the possibility of effective salvage treatment associated with improved survival (online at http://www.oncologiq.nl) - LARYNX.

Primary location Advancement Treatment	↑ Risk upgrade: N status Advanced age Comorbidities Heavy smoking	Group I Very low risk of failure after first-line treatment	Group II Moderate risk of failure after first-line treatment, effective salvage treatment if needed associated with improved survival	Group III High risk of failure after first-line treatment, still possibility of effective salvage treatment associated with improved survival	Group IV Patients not fit for curative intent of recurrence
Larynx, Glottis T1 Surgery/RT		+			
Larynx, Glottis T2 Surgery/RT			+	↑+ ^1^	
Larynx Glottis T3 Surgery/adjuvant RT or RT/CT			+	↑+ ^2^	
Larynx Glottis T4a Surgery/adjuvant RT (RT/CT) or RT/CT					+
Larynx Glottis T4b					+
Larynx, Supraglottis T1 Surgery/RT		+	↑+ ^3^		
Larynx, Supraglottis T2 RT or RT/CT			+	↑+ ^3^	
Larynx Supraglottis T3 RT/CT				+	↑+ ^4^
Larynx Supraglottis T4a; Surgery/adjuvant RT (RT/CT) or RT/CT					+
Larynx Supraglottis T4b					+

Legend:

RT - Radiotherapy alone

RT/CT - Radio chemotherapy

↑- upgrade to the higher group if there are adverse prognostic factors, increasing the risk

^1^Anterior Commissure, Posterior Paraglottic space involvement,

^2^Prior tracheotomy,

^3^N advancement

^4^N advancement, prior tracheotomy

**Table 1b:** HNCs grouped according to the estimated risk of failure after first-line treatment and according to the possibility of effective salvage treatment associated with improved survival (online at http://www.oncologiq.nl) - NASOPHARYNX.

Primary location Advancement Treatment	↑ Risk upgrade: N status EBV negative Advanced age Comorbidities Heavy smoking	Group I Very low risk of failure after first- line treatment	Group II Moderate risk of failure after first-line treatment, effective salvage treatment if needed associated with improved survival	Group III High risk of failure after first-line treatment, still possibility of effective salvage treatment associated with improved survival	Group IV Patients not fit for curative intent of recurrence
Nasopharynx T1-2 N0-N1 EBV -^5^			+		
Nasopharynx T1-3 N2-N3 EBV -					+
Nasopharynx T1-2 N0-N1 EBV +		+			
Nasopharynx T1-3 N2-N3 EBV+			+		
Nasopharynx T4 N0-N3					+

Legend:

RT - Radiotherapy alone

RT/CT - Radio chemotherapy

^5^ Patient treated as negative if the EBV status unknown

**Table 1c:** HNCs grouped according to the estimated risk of failure after first-line treatment and according to the possibility of effective salvage treatment associated with improved survival (online at http://www.oncologiq.nl) – MESOPHARYNX.

Primary location Advancement Treatment	↑Risk upgrade: N status HPV negative Advanced age Comorbidities Heavy smoking	Group I Very low risk of failure after first- line treatment	Group II Moderate risk of failure after first-line treatment, effective salvage treatment if needed associated with improved survival	Group III High risk of failure after first-line treatment, still possibility of effective salvage treatment associated with improved survival	Group IV Patients not fit for curative intent of recurrence
Mesopharynx T1N0-Nl HPV -^6^ Surgery/RT		+			
Mesopharynx T1-2Nl-2 HPV- Surgery/adjuvant RT or RT (RT/CT)			+	↑N2	
Mesopharynx T3-4 N2-N3 HPV- RT (RT/CT)					+
Mesopharynx T1-2 N0-N2 HPV+ RT (RT/CT)		+			
Mesopharynx T3 N0-N2 HPV+ RT (RT/CT)		+			
Mesopharynx T4 N3 HPV+					+

Legend:

RT - Radiotherapy alone

RT/CT - Radio chemotherapy

↑- upgrade to the higher group if there are adverse prognostic factors, increasing the risk

^6^ Patient treated as negative if the HPV status unknown

**Table 1d:** HNCs grouped according to the estimated risk of failure after first-line treatment and according to the possibility of effective salvage treatment associated with improved survival (online at http://www.oncologiq.nl)- HYPOPHARYNX.

Primary location Advancement Treatment	↑Risk upgrade: N status HPV negative Advanced age Comorbidities Heavy smoking	Group I Very low risk of failure after first- line treatment	Group II Moderate risk of failure after first-line treatment, effective salvage treatment if needed associated with improved survival	Group III High risk of failure after first-line treatment, still possibility of effective salvage treatment associated with improved survival	Group IV Patients not fit for curative intent of recurrence
Hypopharynx T1-2 N0-N1 HPV -^6^ Surgery/adjuvant RT or RT/CT			+		
Hypopharynx T3-4 N1-N3 HPV- RT (RT/CT)					+
Hypopharynx T1-2 N0-N2 HPV+ RT (RT/CT)		+			
Hypopharynx T3 N0-2 HPV+ RT (RT/CT)					
Hypopharynx T4 N3 HPV+. RT (RT/CT)					+

Legend:

RT - Radiotherapy alone

RT/CT - Radio chemotherapy

↑- upgrade to the higher group if there are adverse prognostic factors, increasing the risk

^6^ Patient treated as negative if the HPV status unknown

**Table 1e:** HNCs grouped according to the estimated risk of failure after first-line treatment and according to the possibility of effective salvage treatment associated with improved survival (online at http://www.oncologiq.nl) ORAL CAVITY.

Primary location Advancement Treatment	↑Risk upgrade: N status HPV negative Advanced age Comorbidities Heavy smoking	Group I Very low risk of failure after first- line treatment	Group II Moderate risk of failure after first-line treatment, effective salvage treatment if needed associated with improved survival	Group III High risk of failure after first-line treatment, still possibility of effective salvage treatment associated with improved survival	Group IV Patients not fit for curative intent of recurrence
Oral cavity T1 N0-N1 Surgery/RT		+			
Oral Cavity T2 N0-N2 Surgery/adjuvant RT or RT/CT			+		
Oral Cavity T3 N0-N2 Surgery/adjuvant RT/CT				+	
Oral Cavity T3-4 N3 Surgery/adjuvant RT/CT					+

**Table 1f:** HNCs grouped according to the estimated risk of failure after first-line treatment and according to the possibility of effective salvage treatment associated with improved survival (online at http://www.oncologiq.nl).

Primary location Advancement Treatment	↑Risk upgrade: N status Advanced age Comorbidities Heavy smoking	Group I Very low risk of failure after first-line treatment	Group II Moderate risk of failure after first- line treatment, effective salvage treatment if needed associated with improved survival	Group III High risk of failure after first-line treatment, still possibility of effective salvage treatment associated with improved survival	Group IV Patients not fit for curative intent of recurrence
External /Middle ear/Temporal bone T1-2			+		
Temporal bone, lateral skull base T3				+	
Temporal bone, lateral skull base T4					+
Sinonasal/anterior skull base T1-2			+		
Sinonasal/anterior skull base T3				+	
Sinonasal/anterior skull base T4					+

Follow-up visit consisting of 1. ENT examination + neck ultrasound, 2. imaging HN tests, 3. chest imaging, 4. blood tests, and 5. rehabilitation (speech and swallowing) was scheduled with a very different frequency, at the proposed monthly intervals, depending on the group. The number of visits for individual groups varies from 1 to 8 in the first 2 years and from 1 to 17 in the entire 5-year monitoring period. Group IV has not been included in regular follow-up, visits on own initiative of the patient if symptomatic, or supportive care needs, having in mind that third-line therapy and immune checkpoint inhibitors is available (**Tables 1A–F**).

## Discussion

The author’s model assumes a far-reaching differentiation of follow-up schemes depending on the prognosis, risk of recurrence, and chances for potential effective salvage in patients with HNC. What is innovative in this study is the grouping of patients according to the risk of recurrence and the chances of providing effective treatment and not according to the primary location of the cancer. The justification for such construction of the monitoring system is the limited amount of money and human resources that can be allocated to the exponentially growing needs of HNC oncology patients. The proposed model is based on many years of the authors’ own experiences and literature reports with particular emphasis on the evolving global trends in this area.

### Current follow-up models

Numerous medical societies have issued recommendations for monitoring patients with HNC (13, 32, 33), but these have been based mainly on expert opinion rather than solid evidence. Therefore, monitoring schedules were and still are often set arbitrarily by physicians in everyday clinical practice. This approach is associated with excessive frequency of visits or, conversely, too infrequent planning of visits and, secondarily, insufficient sensitivity in detecting recurrences. Both deviations either impose unnecessary economic and financial burdens on healthcare systems or are sometimes ineffective (34). NCCN Clinical Practice Guidelines used to provide a framework for high-value survivorship care for patients with HNC. In general, published guidelines recommend inspection every 1 to 3 months in the first year, every 2 to 6 months in the second year, every 4 to 8 months from the third to the fifth year, and annually thereafter. The number of follow-up visits for 5 years after treatment ranges from 11 to 27 visits (5, 13, 33, 34).

To sum up, patients were usually seen about 15 times over the 5 years. Taking into account the stage of the tumor and overall mortality, this number and timing of follow-up visits according to some authors is adequate for the needs of patients with stage II–IV disease, whereas those with stage I disease may be considered for discharge after the third year if they are told about the risk factors, signs, and symptoms of recurrent disease, and surveillance can be conduct in primary care (35). However, the latest opinions underline that emphasis should be placed on monitoring those patients who can be offered effective salvage (17). The authors have illustrated this regularity and relationship in **Table 2**. Healthcare resources should, therefore, be reallocated in a way that reduces ineffective checkup visits while increasing expenditures and organizing multi-specialty care, including the care of speech therapists, swallowing specialists, dentists, and psychologists for patients with a better chance of recovery and professional activity (17, 36). Patients who are not fit for curative intent of recurrence may receive less intensive imaging, only when turned symptomatic (5, 17). On the contrary, taking into consideration resource allocation patterns and infrastructure density, the therapeutic landscape of locally advanced and recurrent and/or metastatic disease has been rapidly changing with the advent of immune checkpoint inhibitors and better utilization of local approaches (16).

**Table 2:** Proposed universal monitoring algorithm for HNC groups with a high, moderate and low risk of recurrence after the adequate treatment in combination with a high or low probability of using an effective salvage.

Follow-up schedule (months)			3	4	6	8	10	1,2	16	20	24	30	33	36	39	42	45	48	54	60
Group																				
N°	Risk of recurrence	Effective salvage feasible																		
I	Low	yes	1,2		1			1,2,3,4	1	1	1,2,3,4	1		13,4		1		13,4	1	13
II	Moderate	ves	1,2		1,2			1,2,3,4	1,2	1,2	1,2,3,4	1,2	1	1,2,3,4		1		1,2,3,4	1	1,2,3
III	High	yes	1,2	1	1,2	1	1	1,2,3,4	1	1	1,2,3,4	1,2	1	1,2,3,4	1	1	1	1,2,3,4	1	1,2,3
IV	low/moderate/high	no*	1,2		own initiative of patient if symptomatic or needs supportive care															

Legend:

Follow-up visit:

1. ENT examination + neck ultrasound

2. Imaging HN tests

3. Chest imaging

4. Blood tests

5. Rehabilitation (speech, swallowing)

* Check availability of 3rd line therapy and immune checkpoint inhibitors

### Tools for conducting follow-up visits

Tools for conducting patient visits are widely standardized and do not raise controversy. Some centers supplement the classical instrumentation with narrow band imaging (NBI) or high-speed video laryngoscopy, but it is not obligatory.

Traditionally, the follow-up of patients with HNC is clinician-led with lack of standardization in approaches to the Multidisciplinary Team. The identified role of Allied Healthcare Professionals (AHPs) was not only to improve the quality of life (QoL) and symptom control rather than to detect recurrence but also to identify groups who will require more intensive AHP input (37).

Controversy still exists regarding the value of surveillance imaging past the first post-treatment baseline scan in patients who are asymptomatic (1, 38). A decision-analytic Markov model was developed to assess the cost utility of two alternative follow-up programs with a lifetime horizon: program of frequent radiological assessments (maximal approach) compared with a symptom-driven surveillance (minimal approach). In probabilistic sensitivity analysis, 72% of the results lie below the €40,000 threshold (55% below €25,000), and the conclusion has been drawn that an intensive post-treatment follow-up with scheduled radiological assessments over time might be cost-effective compared with symptom-driven surveillance in patients with HNC (39). Similarly, lifetime cost-effectiveness of PET-CT–guided management from a UK secondary care perspective has been proven (40).

Patients with a HNC index tumor have a high risk of second neoplasms located in the lung. In order to achieve an early diagnosis of these SPTs, it would be advisable to establish screening protocols based on the use of low-dose lung CT, which should be maintained indefinitely during the follow-up period (41).

### Specificity of follow-up in different primary HNC locations

When planning the monitoring scheme, up to now, more importance has been attached to the location of the primary and loco-regional tumor advancement and, thus, to the prognosis of survival (35). The key feature of the algorithm presented in this paper is the elimination of divisions into individual primaries in favor of considering patients in terms of the common denominator of the possibility of organ preservation, effective salvage, or inability to undertake salvage treatment. Nevertheless, we discuss some strategic points in organ aspects.

#### Larynx

Despite the small size of the organ itself, the larynx is an extremely heterogeneous primary location, in which the prognosis for cure ranges from 99% for early glottic cancer to a few percent only with advanced processes in the supraglottis at the border of the hypopharynx. An important aspect is the possibility of preserving the organ in early stages of primary and recurrence advancement, in situations where salvage treatment can be carried out using sparing surgical techniques (42–45).

In advanced stages of laryngeal cancer qualified for amputation treatment, the prognosis, role of monitoring, and its effectiveness are completely different (46). The Markov chain model was developed and intended to evaluate the effectiveness of pre-symptomatic detection of breast cancer recurrence, but the model allowed us to make a comparison between the current protocol and various alternatives in larynx cancer survivors (47). Three different follow-up strategies were compared—the current schedule, no follow-up, and the perfect follow-up—in which all recurrences were detected asymptomatically. Compared with no follow-up, the current schedule showed a gain in life expectancy with a range from 0.3 years to 1.5 years that decreased with advancing age. Abolishing the current follow-up schedule raised the disease-specific mortality rate; the increase ranged from 2.8% to 5.9% (48). After total laryngectomy, the prognosis is poor after the development of recurrence. Curative therapy could only be offered to 27.5% of these patients, and only 5% of them were finally disease free (48).

The basis for the diagnosis of vocal fold recurrence is endoscopic examination with stroboscopy as the gold standard (49). However, there are some limitations associated with conventional white light imaging together with stroboscopy in terms of laryngeal cancer follow-up. Application of NBI, as one of biological endoscopic evaluation, has created a new direction due to filtering of microvessels, which indicate neoangiogenesis. This technique enabled the assessment of the pathological vessels in previously treated cancerous laryngeal mucosa and detected recurrence early during the follow-up, especially in a difficult-to-visualize site such as the hypopharynx. On the other hand, an additional tool to stroboscopy is high-speed videoendoscopy, which is an accurate method for an objective assessment of vocal fold oscillations (50).

#### Oral cavity

In oral cavity, salvage treatment saves only 22% of patients with recurrent disease. Over half of patients with recurrence were detected at pre-scheduled follow-up visits; minority were detected at extra visits at the patients’ own request. The necessity and cost-effectiveness of a routine follow-up schedule can thus be questioned, given that there is a very limited effect on survival. After 2 years, follow-up should be tailored to the individual needs of patients for supportive care and monitoring of late side effects of the treatment (19, 51).

Surveillance imaging is critical during follow-up period to detect recurrence in oral cavity cancer for effective salvage surgery. MR imaging has potentially high value to detect recurrent tumor especially after primary surgery without flap reconstruction up to developed new technical application as T2 weighted image (T2WI) in Diffusion-weighted magnetic resonance imaging (DWI or DW-MRI) technique that could be more sensitive with flaps surgery (52). Fluorodeoxyglucose (FDG)-PET seems to be the most reliable tool for loco-regional surveillance of patients after resections with flap reconstruction (53).

#### Pharynx

Regardless of the location of the malignancy in the nasopharynx, oropharynx, or hypopharyx and its histological type, the postoperative care is similar and consists of head and neck examination including videoendoscopy and dental assessment of the oral cavity and areas exposed to radiotherapy. Epstein-Barr Virus (EBV) DNA monitoring in the blood should be considered in nasopharyngeal carcinoma, but the clinical benefit of this is not defined. In addition to the above general guidelines for all areas of the pharynx, the location of a neoplasm in the oropharynx specifically requires consideration of HPV testing. Some centers may recommend HPV testing during follow-up visits to assess the persistence or clearance of HPV infection, which can help inform prognosis and guide further management decisions.

The recurrence of hypopharyngeal cancer is a high-risk of fatal disease that is associated with poor prognosis and high risk of complications due to salvage treatment. Complication rate is higher in salvage mainly because of high percentage of prior chemotherapy and generally poor outcome of hypopharynx cancer (11, 54, 55). A 5-year overall survival (OS) of 27% for salvage (pharyngo)laryngectomy after primary chemoradiation was observed (55). As was reported the mean time to recurrence before salvage surgery was 7.5 months (56).

Oropharyngeal squamous cell carcinomas 5-year OS oscillated between 21% and 44%, but, nowadays, it is mostly dependent on HPV-positive or HPV-negative disease (57). HPV-positive tumors generally have a more favorable outcome, but still about 10% of patients could experience (loco)regional failure. Nevertheless, HPV-positive oropharyngeal squamous cell carcinomas (OPSCCs) do have a better outcome in salvage surgery (58). The recurrence-free survival is longer in HPV-positive tumors and OS in HPV-positive patients with disease progression after locoregional failure and after salvage surgery is more satisfactory (58).

Recurrent nasopharyngeal carcinoma is highly challenging option for treatment. Early detection of recurrence is mandatory. Salvage surgery is an option for carefully selected patients with a local recurrence (59, 60). The survival curves are distinctly separated within these stages [5-year OS of 72.0%, 55.1%, 21.1%, and 10.1% for surgical stages I, II, III, and IV, respectively (61)]. Three-year survival rates up to 60% after salvage surgery was observed mostly in T1 and T2 recurrent disease. Advanced stages with skull base, cranial nerve, and dural or brain involvement are associated with poor prognosis (62). It has been proven that surgical salvage may be superior to re-irradiation using intensity modulated radiation therapy (IMRT) (re-IMRT) in terms of laryngeal cancer (LC), OS, and QoL. Surgical salvage treatment is associated with significantly better OS compared with re-IMRT (5-year OS of 77.1% vs. 55.5%) (61, 62).

#### Salivary glands

The basic, cheap, readily available, and widely accepted tool for the assessment of the glands, and the bed after surgery of the benign salivary tumors is ultrasonography. Its only limitation is the depth of the tissues that can be assessed, so, in some cases, it should be replaced with MRI (63). In malignant tumors, a follow-up consisting of contrast enhanced MRI should be supplemented.

##### Salivary gland benign tumors

Majority of benign salivary gland tumors are located in the parotid gland with PA being the most frequent. Recurrence of PA after surgical treatment is reported in the literature at 2-8% (64, 65), and its malignant transformation is estimated at up to 6% (66). Risk factors for PA recurrence are capsule rupture, tumor spillage, margin status, satellite tumors, histological subtype, deep lobe location, younger age at diagnosis, and subsequent recurrence, and these cases should be included into group requiring more thorough follow-up (66, 67). PA recurrence in the parotid gland is often multinodular; thus, its early detection is essential to provide save for the facial nerve treatment (66). Secondary malignant rate (carcinoma ex PA in recurrent PA) is low, estimated in the published reports for 0% to 23%, and it may increase with number of recurrences, time, and microscopic severe dysplastic features (68).

Considering the above risk factors for recurrence and the potential for malignant transformation, patients after PA surgery should be divided into two groups: low and increased risk of recurrence. Patients with a low risk of local recurrence may self-examine (self-monitoring), and ultrasound examination of the operated area performed once a year. In the group with an increased risk of local recurrence, in addition to self-monitoring, in the first year after treatment, an ultrasound examination every 6 months, in subsequent years every 12 months, depending on the primary location, replacing it with an MRI examination should be considered.

##### Salivary gland malignant tumors

Salivary gland malignant tumors (SGMTs) are very heterogenous group (69), and they have different clinical course; more than 20 histological types have been distinguished, which may additionally be characterized by a low-grade, intermediate, high-grade, and variable grade of malignancy (Nishida). In addition to the histological structure, the prognosis is influenced by the primary location of the tumor (parotid, submandibular, sublingual gland, and minor salivary glands), sex, and patient’s age. In more than 50% of salivary malignancies, the perineural spread is observed, and this feature increases the risk of recurrence. Distant metastases are the most frequently located in lungs (80%) followed by bones (15%) (70). The best imaging technique for diagnosis of the primary SGMTs and for their follow-up is MRI (63). Recurrence of SGMT can occur many years after initial treatment; therefore, follow-up longer than 5 years is necessary.

Taking into consideration all described risk factors, we propose a follow-up consisting of contrast enhanced MRI every 6 to 12 months in the first 2 years after treatment, every 12 months in the subsequent years, and chest CT scan every 12 months. Initial for follow-up MRI should be performed 3 moths after the treatment is completed.

Adenoid cystic carcinoma is considered to be a high-grade cancer, and, with its tendency to perineural spread, it requires strict follow-up: MRI every 3 to 4 months in the first 2 years of follow-up, every 6 months during next 3 years, and every 12 months in subsequent years (69). Chest CT scan should be performed every 12 months as in other SGMTs. Because of its slow growth and high risk of recurrence and metastases many years after initial treatment, the lifelong follow-up is recommended.

#### Sinonasal, temporal bone, and skull base

Malignant neoplasms of the temporal bone constitute a small percentage of all HNC (only 0.2%), and the local advancement of the process is associated with a poor prognosis (71, 72). Local recurrences amount to 32.3% (73). The average rate of hidden metastases in the region II of the neck is 14%; in the local advancement, T3 is estimated at 21%. Considering the high rate of recurrences in the early postoperative period, follow-up should include a physical examination and MRI performed every 2 months in the first year, every 4 months in the second year, and every 6 months from the third to the fifth year (73, 74). Molecular markers are not yet significant in the diagnosis and follow-up of patients with temporal bone cancer (75). In follow-up, the need for auditory rehabilitation should be taken into account, with potential use of bone-integrated hearing implants (76).

Sinonasal malignancy surveillance strategies may warrant modifications of current protocols used for HNC. This is due to several factors, including a greater diversity of tumor histology and duration of post-treatment sinonasal inflammation (77, 78). Sinonasal cancer tends to exhibit a higher rate of local failure and occur in a delayed fashion compared with mucosal HNC. Moreover, the site of failure and time-varying risk of recurrence is histology-specific (79). Endoscopy has low sensitivity in recurrence detection, especially in the asymptomatic patient; CT, MRI (Khalili), and PET/CT (Ozturk) are useful although prolonged inflammation can lead to a high number of false positives (77). Following multimodality treatment of the skull base, patients may experience endocrine, visual, auditory, sinonasal, olfactory, and neurocognitive deficits, resulting in poor QoL. Thus, patients with sinonasal cancer would benefit from tailored survivorship programs to address not only the recurrence but also functional impairments resulting from disease and treatment toxicity (79). Regardless of the type of imaging, symptomatic presentation after treatment had a specificity of 91.0%, and the frequency of scans was not associated with the risk of recurrence. For the symptomatic presentation to be strongly associated with recurrence, patients should be investigated with appropriate imaging at presentation. This last research was in line with our conclusions. In the schemes proposed by the authors, patients with sinonasal and temporal bone cancer who underwent radical surgery with adjuvant RT are assigned to the fourth group.

#### The role of monitoring in side effects of therapy diagnostics and management

Follow-up has a significant impact on the timely management of the consequences and complications of cancer treatment. This extremely broad topic is not the subject of this study; however, we will list the most important issues.

Oncological treatment with chemotherapy or biological therapies causes a number of side effects and dysfunctions that affect the general health and QoL of patients. These include insufficiency of endocrine glands, central and peripheral nervous system, deafness, or atrophic mucositis.

Hypopituitarism after skull base radiotherapy has to be emphasized. Serum levels of cortisol, growth hormone, free T4, prolactin, insulin-like growth factor 2, luteinizing hormone, folliculotropic hormone, adrenocorticotropic hormone, and total and bioavailable testosterone should be annually tested. Thyrotropic hormone (TSH) alterations may indicate thyroid dysfunction or hypopituitarism in skull base irradiation patients (4). The need to monitor thyroid function is emphasized for elevated TSH levels that have been detected in 20%–25% of patients who have received neck irradiation; thus, TSH should be tested every 6 to 12 months in this group (13).

Oral cavity, oropharynx, and hypopharynx treatment influence the swallowing; thus, evaluations by a speech-language pathologist may be recommended to assess any swallowing and provide appropriate interventions if needed. Regular dental checkups and oral hygiene maintenance are important to manage any treatment-related dental and mucosal issues. Nasopharyngeal cancer treatment can affect hearing function; thus, regular otology evaluations are recommended to monitor conductive hearing loss and provide appropriate intervention if needed (13).

Laryngopharyngeal cancer treatment can impact voice quality and function; thus, speech-language pathologist evaluations are recommended to assess any voice changes and provide appropriate interventions if needed.

As the adjuvant radiotherapy is frequently applied in HNC treatment, the risk of radiation-induced malignancies in the head and neck region is increased. Therefore, like in each irradiated patient, the otorhinolaryngological examination, including endoscopy, should be performed every 1 to 3 months in the first year of follow-up, every 2 to 6 months in the second year, every 3 to 4 months in the years 3 to 5, and every 12 month in the next years (4).

Summarizing, the follow-up of patients with HNC should be multidisciplinary because of consequences of the primary tumor extent and treatment given. The importance of involving various specialists and coordinating care among them capture the complexity of optimal follow-up care in patients with HNC and include mainly radiotherapists, chemotherapists, and surgeons, and, in selected cases, endocrinologists, gastroenterologists, audiologists, gastroenterologists, dentists, peridontologists, and others.

### Patients’ preferences regarding follow-up

Approximately 25% of patients disengaged from important follow-up care within 1 year. Lack of social support, depressive symptomatology, and single-treatment modality (80); unmarried status; a longer driving distance to the facility (although rurality was not associated with discontinuation); nor age, gender, or payer status (81) may be important correlates of discontinuation of care in patients with HNC. Other reported reasons of lost to follow-up are to enlist: 22% had difficulty scheduling an appointment, 30% had transportation barriers, 22% had personal or work obligations that prevented follow-up, 17% did not follow-up because they “felt better,” and 39% were following up with an otolaryngologist or oncologist closer to home (3). In addition, overall adherence to the American Cancer Society HNC Survivorship Care Guideline (82) in early post-treatment survivors occurred to be suboptimal (Salley JR). Understanding these barriers is critical to creating a patient-centered model that balances both clinical surveillance needs and reasonable expectations for patients. Improvements can be made to educate patients on the recommended length of follow-up and its importance (3).

Patients’ preferences regarding follow-up remain poorly investigated. The cross-sectional survey revealed that 89.1% preferred scheduled follow-up to self-referral, 57% favored fewer visits than the current standard, and 85.1% endorsed regular imaging. Moreover, patients’ preferences only partially correspond to current follow-up guidelines (83).

Patients’ preferences before and after treatment are closely connected with QoL. Improving it in patients with HNC is a very vast problem and exceeds the scope of this article. Although, it should be mentioned that existing literature for intervention studies focuses on educational, psychosocial, physical, and psychological symptom management; mindfulness; pharmacology; exercise; and telemedicine strategy. Appearance of HNC recurrence exacerbates the problem. However, using therapeutic coping mechanisms to control discomfort during and after treatment can affect mood and QoL well into the survivorship stage (84, 85).

### HNC follow-up scheme optimization

Risk stratification–guided surveillance based on retrospectively collected local and distant recurrence of chemoradiation treated patients was presented in 2016 (86).

Personalized follow-up program (PFU) that provides a potential alternative to the standardized intense follow-up schedule was presented for all patients with HNC in 2022. PFU followed the NHS Long-Term Plan for Cancer, where each patient moved to a follow-up pathway that suits individual needs through personalized stratified follow-up programs (NHS England, 2019; https://doi.org/10.1016/j.annonc.2022.07.401).

PETNECK2 is a program of research (NIHR200861) with an embedded randomized controlled trial designed to determine whether patient-initiated follow-up (PIFU) is more effective than regular follow-up for HNC. PETNECK2 builds on the successful results of the first PETNECK study, where a 3-month PET-CT scan reliably detected patients requiring neck dissection, avoiding unnecessary surgery for those at low risk of recurrence, and reducing harm and costs (87). Current PETNECK2 trial will compare a new model of PIFU with routine scheduled follow-up. UK clinicians were enthusiastic about the PETNECK2 trial but had concerns that PIFU may not suit disengaged patients and may aggravate patient anxiety/fear of recurrence and delay detection of recurrence (88).

The next Individualized Follow-Up for Head and Neck Cancer (INFLUENCE) study offered a decision-aided choice between standardized or individualized follow-up after 1.5 years of uncomplicated guideline-prescribed follow-up. Standardized follow-up entails continuing the 5-year guideline-prescribed schedule. Individualized follow-up means the patient only attends the outpatient clinic on their own initiative in case of physical symptoms or supportive care needs (15). Patients are educated on self-examination and when a control visit is necessary (6). Moreover, the same authors point out that 1 year of follow-up for oral cavity squamous cell carcinoma (SCC) and 1.5 years for oropharynx-, larynx-, and hypopharynx SCC suffice for the goal of detecting disease manifestations after treatment (6).

A pilot-tested clinical informatics intervention, HN-STAR, was developed to elicit concerns online from HNC survivors prior to a routine oncology clinic visit. HN-STAR then presents tailored evidence-based clinical recommendations as a clinical decision support tool to be used during the visit where the oncology clinician and survivor select symptom management strategies and other actions. This generates a survivorship care plan (SCP). Online elicitation of health concerns occurs 3, 6, and 9 months after the clinic visit, generating an updated SCP each time. HN-STAR encompasses important methods of improving survivorship care (89).

### HPV and EBV as a game changers in HNC surveillance strategies

HPV or EBV status is rarely considered when determining surveillance plans. In particular, HPV+ and HPV− OPC are two distinct entities that require 4 and 12 follow-up visits, respectively (17). The presented current findings reveal an urgent need for individualized surveillance strategies in patients with HNC cancers’ HPV- and EBV-based etiology. Thus, a patient-tailored assessment plan should integrate prognostic factors based on HPV or EBV positivity (90). However, overlapping high-risk features may necessitate a modification of the recommended follow-up visits, for example, in favor of more frequent visits for patients with HPC+ OPC with a smoking history of more than 10 pack years (17).

## Conclusions

The presented algorithm summarizes HNC follow-up problems. This paper is also a call to action for implementing the recommended strategies. We wanted to highlight areas where further research is needed to refine follow-up care in HNC.

Multidisciplinary cooperation and the development and refinement of follow-up recommendations by the experts of the individual scientific societies are essential. The need for more specific recommendations stems from the specificity of the primary tumor site, considering the histological specificity of the tumor.

Novel approaches and technologies in follow-up care should be explored in more depths, i.e., established visualization technique such as biological endoscopy (NBI), and liquid biopsy should be integrated into the standard practice with all potential benefits and challenges.

Universal monitoring algorithm for HNC four groups with a high, moderate, and low risk of recurrence after the adequate treatment in combination with a high or low probability of applying an effective salvage is an absolutely innovative approach to redeploying system resources and ensuring maximum benefit for patients with HNC.

The division of all HNCs into four different monitoring paths ensures standardization of procedures, repeatability of the scheme, and redistribution of forces and resources to groups that can get the greatest therapeutic benefit from dense meshes of the visits network.

The presented algorithm practically eliminates from follow-up visits a very large group of the most advanced patients, with no chance for further curative treatment. Nevertheless, intensive palliative support should be planned for this group in dedicated places, which should be the subject of another study.

## Data availability statement

The original contributions presented in the study are included in the article/supplementary material. Further inquiries can be directed to the corresponding author.

## Author contributions

MW: Conceptualization, Formal Analysis, Investigation, Methodology, Supervision, Writing – original draft, Writing – review & editing. JM: Conceptualization, Writing – original draft, Writing – review & editing. WP: Funding acquisition, Project administration, Writing – original draft, Writing – review & editing. PB: Writing – original draft, Writing – review & editing. BM: Writing – original draft, Writing – review & editing. MR: Writing – original draft, Writing – review & editing. KS: Writing – review & editing. PM: Writing – original draft, Writing – review & editing. JF: Writing – review & editing. DJ: Writing – review & editing. KN: Writing – review & editing. AM: Writing – original draft, Writing – review & editing.
